# Glucocorticoid Signaling and Epigenetic Alterations in Stress-Related Disorders

**DOI:** 10.3390/ijms22115964

**Published:** 2021-05-31

**Authors:** Niki Mourtzi, Amalia Sertedaki, Evangelia Charmandari

**Affiliations:** 1 Division of Endocrinology, Metabolism and Diabetes, First Department of Pediatrics, “Aghia Sophia” Children’s Hospital, National and Kapodistrian University of Athens Medical School, 11527 Athens, Greece; nikimourtzi23@gmail.com (N.M.); aserted@med.uoa.gr (A.S.); 2 Division of Endocrinology and Metabolism, Center of Clinical, Experimental Surgery and Translational Research, Biomedical Research Foundation of the Academy of Athens, 11527 Athens, Greece

**Keywords:** stress response, glucocorticoids, epigenetics, stress-related disorders

## Abstract

Stress is defined as a state of threatened or perceived as threatened homeostasis. The well-tuned coordination of the stress response system is necessary for an organism to respond to external or internal stressors and re-establish homeostasis. Glucocorticoid hormones are the main effectors of stress response and aberrant glucocorticoid signaling has been associated with an increased risk for psychiatric and mood disorders, including schizophrenia, post-traumatic stress disorder and depression. Emerging evidence suggests that life-stress experiences can alter the epigenetic landscape and impact the function of genes involved in the regulation of stress response. More importantly, epigenetic changes induced by stressors persist over time, leading to increased susceptibility for a number of stress-related disorders. In this review, we discuss the role of glucocorticoids in the regulation of stress response, the mechanism through which stressful experiences can become biologically embedded through epigenetic alterations, and we underline potential associations between epigenetic changes and the development of stress-related disorders.

## 1. Introduction

Stress is defined as a state of threatened or perceived as threatened homeostasis. A broad spectrum of extrinsic or intrinsic, real or perceived stressful stimuli, called ‘stressors’, activates a highly conserved system, the ‘stress system’, which adjusts homeostasis through central and peripheral neuroendocrine responses. The stress response depends on highly complex and tightly-regulated processes and involves the cross-talk of molecular, neuronal and hormonal circuits [1]. The well-tuned coordination of these systems is indispensable for an organism in order to adapt to stressors and re-establish homeostasis (adaptive stress response) [2].

In mammals, glucocorticoid hormones are the main central effectors of the stress response, playing a crucial role in the effort of an organism to maintain its homeostasis during periods of stress [1]. Aberrant glucocorticoid signaling in response to stressors can lead to sub-optimal adaptation of an organism to stress events, a state that has been defined as cacostasis or allostasis [2]. When an organism falls into cacostasis, it strives to find ways to survive and maintain its stability outside of the normal homeostatic range, and the cost that it has to pay for this purpose is termed allostatic or cacostatic load [2].

Emerging evidence suggests that exposure to early-life stress events may alter the epigenomic landscape, which is defined as changes in the regulation of gene expression without changes to the DNA sequences [3]. The long-lasting nature of epigenetic changes led the scientific community to propose that glucocorticoid secretion in response to stress, as well as genes involved in the glucocorticoid signaling pathway, play a fundamental role in shaping a form of epigenetic memory through which stressful experiences become biologically embedded [3]. Critically, transcriptional dysregulation caused by aberrant epigenetic changes in glucocorticoid-related genes has been pathophysiologically linked with the emergence of a host of stress-related disorders [4].

The current review provides an update on the (a) molecular mechanisms of glucocorticoid action and the mechanisms through which glucocorticoid signaling mediates stress response; (b) the glucocorticoid-induced epigenetic changes on genome integrity during stress; and (c) existing evidence that links glucocorticoid-interceded epigenetic alterations with stress-related disorders.

## 2. Glucocorticoid Signaling via GRs and MRs upon Stress Response

**Figure 1:**
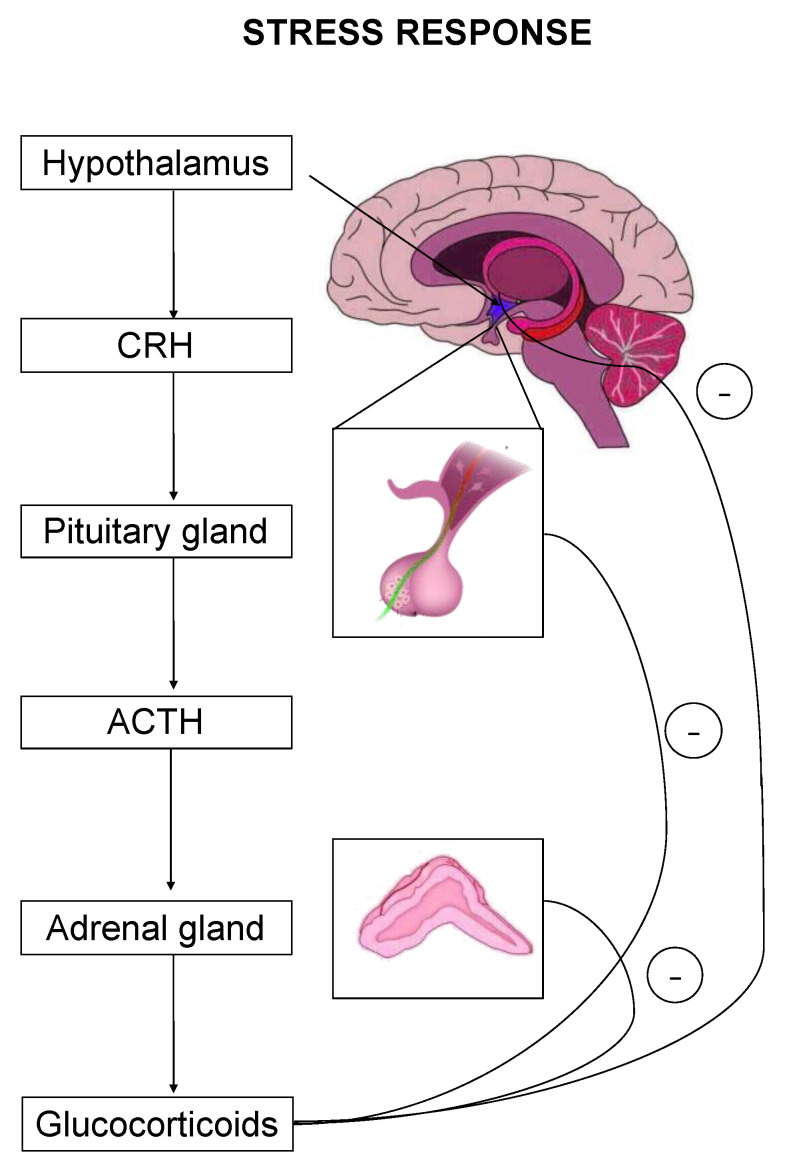
The hypothalamic–pituitary–adrenal (HPA) axis is the primary effector of a stress response. Critical hormones and organs are shown, whereas cycles with the negative symbol indicate negative feedback mechanisms.

The stress response is primarily mediated by the hypothalamic–pituitary–adrenal (HPA) axis-, as well as by effectors acting on peripheral organs [2,5,6,7]. Upon stress-induced activation, the HPA axis stimulates the release of adrenocorticotropic hormone (ACTH) that drives the adrenal secretion of glucocorticoids in the blood circulation [8] (Figure 1). Glucocorticoids exert their effects on target organs mainly through binding and activation of two types of receptors, the mineralocorticoid (MR) and the glucocorticoid (GR) receptor [1].

On the one hand, glucocorticoids can have a generalized impact on multiple organs through binding to GRs that are abundant in almost every mammalian tissue, through genomic and nongenomic mechanisms [9]. Regarding the genomic-dependent pathway, GRs can regulate the expression of a wide variety of genes either through direct binding in glucocorticoid response elements (GREs) of GC-responsive genes [10] or through physical interaction with transcription factors [11]. In contrast to the genomic actions of GC that occur within hours or days [12], GRs can also exert rapid actions, which take place within seconds or minutes, through nongenomic mechanisms that do not involve GRs transcriptional activity and are not mediated by the genome [12]. The nongenomic actions of GCs are primarily mediated via membrane GRs (mGRs), a different isoform of the classic GR (termed as GRγ) that trigger the activation of kinase signaling pathways, such as the mitogen-activated protein kinase (MAPK) or the phosphatidylinositol 3-kinase (PI3K) cascades [13,14,15]. Moreover, GCs can directly influence the ion transport process by interlocating into the membranes of target cells [16] or bind and alter the activity of membrane receptors (such as ion channels, G-protein coupled receptors, enzyme-linked receptors) [13,14,15]. Examples of nongenomic GCs action during the stress response include rapid feedback inhibition of ACTH secretion from the anterior pituitary through mGR 28, regulation of basal intracellular Ca^2+^ homeostasis29 and activation of ERK1/2 MAPK pathway, possibly through mGR action29.

On the other hand, MRs are predominantly expressed in limbic areas (hippocampus, amygdala and prefrontal cortex) together with GRs [17], and are characterized by a 10-fold higher binding affinity for glucocorticoids than GRs [18]. Due to the higher occupancy rate of MRs, it is thought that MRs play a more critical regulatory role in stress reactivity by setting the threshold of the HPA axis activation [19] rather than having an autonomous role as the main stress effector. Similar to GRs, the effects of GCs are mediated through MRs via genomic and nongenomic pathways [19]. MRs, upon GC binding, can act as transcription factors and alter the expression of several stress-associated genes, such as FK506 binding protein 5 (*FKBP5*) [20] glucocorticoid-induced leucine zipper (*GILZ*) [21], period circadian clock 1 (*PERL1*) [22] and serum/glucocorticoid regulated kinase 1 (*SGK1*) [23]. The rapid nongenomic action of MRs is mediated through membrane-bound MR (mMR) [24], including glutamate release from hippocampal CA1 neurons [25] and enhancement of glutamatergic transmission in basolateral amygdala neurons (BLA) [26].

## 3. Functional Synergistic Network of MR and GR

MRs and GRs interact with each other throughout the stress response, mediating distinct but complementary roles, and their synergistic action is considered essential for the maintenance and/or restoration of homeostasis. Interestingly, despite the fact that GRs and MRs recognize the same “GRE” sequence, their target genes, partially overlap and their effects may be different and sometimes opposites [27]. MRs are responsible for the appraisal of new information, activation of the stress reaction and memory retrieval of previous stress-associated strategies. Moreover, the genomic actions of classic MRs are responsible for setting the HPA axis sensitivity or threshold of the stress response [28].

**Figure 2:**
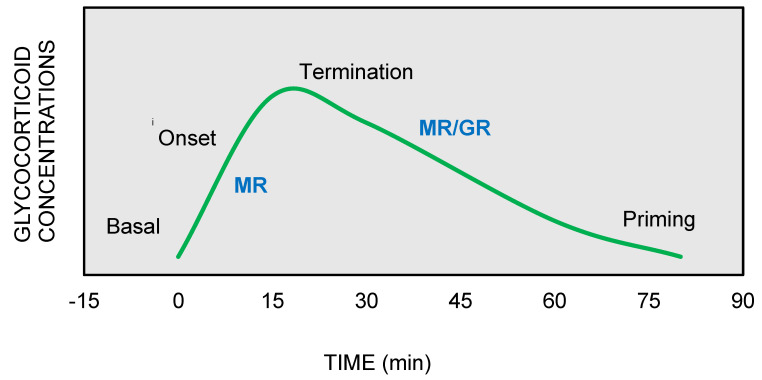
The stress response can be divided into four phases: (**1**) Basal: intracellular MRs are occupied with low concentrations of glucocorticoids (GCs). (**2**) Onset: GCs secretion from the adrenal cortex in response to a stressful event. GCs bind to MRs, which are progressively activated as the concentrations of GCs increase (**3**) Termination: GRs are activated and mediate the termination of stress response by exerting negative feedback at the hypothalamic and anterior pituitary level (**4**) Priming: Stressful experiences will be stored through GRs action in hippocampus and prefrontal context for future use.

On the other hand, GRs are indispensable for the termination of stress response, the energy mobilization, the recovery, and the memory storage of stressful experiences for future purposes. It has been suggested that the stress response could be divided into four phases, based on spatiotemporal features of nongenomic and genomic GCs actions [27,29,30] (Figure 2).

Phase 1—Basal: At the basal state, intracellular MRs are occupied with low, nonstress concentrations of GCs, owing to their strong GC-binding affinity. The secretion of GCs following a stress event allows the additional binding of GCs to the lower-affinity GRs, which are progressively activated as the concentrations of GCs are further increasing [31]. Thus, the constant GC occupation of MRs determines the threshold for activation of stress response, while the short-term activation of GRs provides flexibility to the stress system to respond to sudden GC changes as they occur [32].

Phase 2—Onset: When a stressor factor that threatens homeostasis or perceives to do so appears, the hypothalamus releases CRH that triggers GC secretion from the adrenal cortex. Instantly, mMRs, through nongenomic actions, increase the levels of attention and vigilance in support of recognizing and assessing the risk of the new threat [33]. At the same time, the mMR-induced excitability of hippocampal neurons permits the memory retrieval of previous stress-coping strategies [34], while in the amygdala region, the fight-and-flight response is activated though MRs actions that promote feelings of fear and anxiety [34,35]. Critically, the limbic MR activation determines the stress-coping style that is deployed, with mild-stress stimuli promoting a “thinking” strategy, which is conducted in the hippocampus, instead of a “doing” strategy that involves increased activation of the amygdala.

Phase 3—Termination: As the concentrations of GCs progressively increase during the stress response, low-affinity GRs become active and they exert negative feedback at the level of the hypothalamus and anterior pituitary, and contribute to the termination of stress response [36]. The termination is achieved through transcription-independent and transcription-dependent GR actions that mediate (a) fast negative feedback at the pituitary (ACTH inhibition) and hypothalamic levels (CHR inhibition) (<10 min) [37], followed by (b) intermediate negative feedback acting on the paraventricular nucleus (PVN) of the hypothalamus (30 min–2 h) and (c) slow and long-lasting negative feedback (>3 h) that involves suppression of pro-opiomelanocortin (POMC) expression, the ACTH precursor molecule [38]. In parallel, through transcriptional regulatory mechanisms, the GRs transmit the signal to multiple organs for the mobilization of energy resources in order to meet the energy demand of recovery to basal state [39].

Phase 4—Priming: The stressful experience, along with its selected coping style, is encoded and stored in the hippocampus and prefrontal context through the GRs actions for future use [40,41]. Memory consolidation of stress experience enables MR-mediated memory retrieval when similar situations arise in the future. During the priming stage, the GRs induce the activation of NF-κB NLRP3 inflammasome pathway [42], which may potentiate the neuroinflammatory response to a subsequent pro-inflammatory challenge [43]; this mechanism has been proposed to explain the paradoxical proinflammatory actions of GCs, traditionally considered as anti-inflammatory agents, observed in some clinical conditions [44].

Thus, MRs and GRs form a tightly co-regulated network that coordinates stress response and this observation led to the genesis of MR/GR balance hypothesis [30]. According to this hypothesis, an imbalance between the effects of GRs and MRs during the stress response may lead to dysregulation of the HPA axis and an inability to adapt to stressors and restore homeostasis, thereby conferring an increased vulnerability to a number of stress-related disorders [45].

## 4. Epigenetic Alterations as a Cause of Dysregulation of the Stress Response

An important mechanism that has been implicated in the development of MR/GR imbalance and HPA axis dysfunction pertains to the epigenetic alterations induced by major life stressors [46]. Epigenetics encompasses all the mechanisms influencing gene expression, without any change in DNA sequences [4], and is viewed as the interface between the genome and the environment [47]. These include DNA methylation, post-translation histone modifications, noncoding RNAs and three-dimensional changes in chromatin conformation [47]. It has been proposed that epigenetic modifications can be a part of a memory system that stores and transmits information about past stressful experiences to progeny cells, thus shaping cellular responses to subsequent stressor stimuli [46].

Regarding the mechanism linking epigenetic regulation with the stress response, it has been suggested and demonstrated through several functional studies that the GR activation by GCs instigates not only changes in gene transcription upon stress, but also alterations in DNA methylation patterns, with the most preeminent change being the DNA demethylation at or near the GRE elements [3].

**Figure 3:**
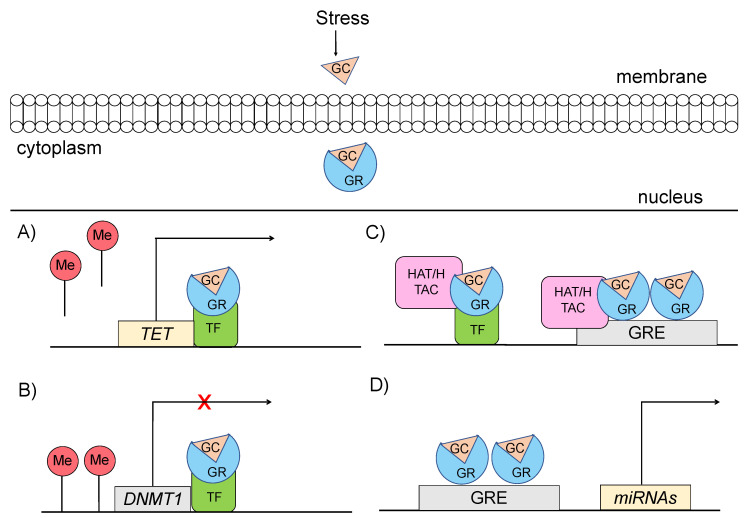
Mechanisms through which glucocorticoid-mediated activation of GR can induce epigenetic changes (**A**) GRs through interaction with transcription factors (TF) catalyze active demethylation of enzymes involved in demethylation processes, such as the TET family of 5-methylcytosine dioxygenases. (**B**) GRs catalyze active methylation of enzymes involved in methylation processes, such as DNMT (DNA methyltransferase 1, DNMT1). (**C**) GRs activation can induce histone modifications via direct GR binding on GRE elements or via interaction of GRs with TFs that recruit histone acetyltransferases. (**D**) GRs activation can regulate the expression of microRNAs, enriched in GRE elements. GC: glucocorticoid, GR: glucocorticoid receptor, GRE: glucocorticoid response elements, DNMT1: DNA methyltransferase 1, HAT: histone acetyltransferases, HDAC: histone deacetylase, ME: methionine, TET: ten-eleven translocation methylcytosine dioxygenases, TF: transcription factor.

Specifically, glucocorticoids can shape the epigenome through four major mechanisms, which primarily involve GR signaling (Figure 3) [48,49]: (a) GCs can rapidly and dynamically evoke demethylation of cytosine-guanidine dinucleotides (CpGs) at or near GREs [3]. The mechanism behind this action implicates GC-dependent transcriptional upregulation of enzymes that catalyze active demethylation (ex. TET family of 5-methylcytosine dioxygenases), as well as downregulation of DNA methyltransferases (ex. DNA methyltransferase 1, DNMT1) [50]. Although GR signaling during stress has been generally associated with demethylation processes, the action of GRs has also been linked with methylation of a number of genes [51]; (b) GCs can induce histone modifications, such as methylation and acetylation of histone proteins, via direct GR binding or via interaction of GRs with other transcription factors (TFs) that recruit histone acetyltransferases [3]. For instance, the TF RelB/p52 has been shown to recruit CBP binding protein and HDAC deacetylase that promote acetylation and deacetylation of histone 3 Lys in the promoter regions of *CRH* and *COX-2* genes [52]; (c) GCs can regulate expression patterns of several miRNAs, such as miR-218, miR-124, miR-29a, possibly through GR action, given that the genes encoding the altered miRNAs are enriched in predicted as GRE sites [53]; and (d) GCs can lead to chromatin remodeling via GR-activation, changing the accessibility of genomic regions to TFs [54].

Epigenetic alterations induced by GCs can appear at specific genomic loci but also at the genome-wide level [3]. For instance, prenatal exposure to untreated maternal depression has been linked with lower DNA demethylation patterns across the genome [55]. At the gene-specific level, epigenetic changes in genes involved in the HPA axis stress response pathway, have been consistently reported in a number of studies. In the context of stress responses or stress-related disorders, the most extensively studied epigenetically altered genes are the *FKBP5*, *NR3C1*, *BDNF*, *SLC6A4* and *OXTR* [56,57].

Convergent evidence suggests that exposure to stress leads to hypomethylation of the gene encoding *FK506 binding protein 51*, *FKBP5*, which acts as a negative regulator of the GR signaling cascade [58]. In particular, it has been shown that FKBP5 interacts via the heat-shock protein HSP90 with GR and reduces the affinity of GR for binding to GCs by altering the folding of GR. Through this mechanism, FKBP5 suppresses the GR nuclear translocation and the concomitant action of GR as a transcription factor [59]. Thus, hypomethylation of *FKBP5* has been proposed to constitute a negative feedback mechanism that constrains the activity of the HPA axis [59]. Critically, *FKBP5* is characterized by tissue expression specificity, with high baseline expression levels of the FKBP5 protein observed in the hippocampus but low levels in other areas, such as the hypothalamus [59]. The context-dependent expression of *FKBP5* has been shown to influence the degree of Fkbp5 mRNA induction following GC administration in mouse models [60]. In particular, regions characterized by low levels of FKBP5 protein at baseline (hypothalamus, amygdala) showed a higher fold-change increase in *FKBP5* mRNA expression levels compared to regions with high FKBP5 baseline expression levels (hippocampus) [60]. The context-dependent expression of FKBP5 that dictates the tissue-specific response to stressors should be taken into account when we consider *FKBP5* dysregulation as a risk factor for development of stress-related disorders and when we assess the effects of drugs inhibiting *FKBP5*, as we will discuss later.

The GR gene, *NR3C1*, has been extensively studied in the context of epigenetic regulation of the stress response. Findings from animal models, replicated in humans, point towards a stress-induced increased level of *NR3C1* methylation that has been associated with decreased levels of *NR3C1* expression [61]. Stress-induced epigenetic modification of the *NR3C1* gene occurs across the lifespan, from prenatal life [3,7] to late adulthood [62], with the early and later life periods being more sensitive to stressors given that in these periods, we observe either rapid or less efficient epigenetic adaptation, respectively [3]. Critically, it has been demonstrated that methylation changes occurring early in life can have long-term effects, impacting the plasticity of stress response mechanisms and jeopardizing the adaptation to future stressful situations [63].

*BDNF* encodes the brain-derived neurotrophic factor that contributes to the neuronal plasticity of the brain by regulating the activity of several neurotransmitter receptors, such as GABA and tyrosine kinase B (TrkB) receptors, and plays a crucial role in learning and memory formation [64]. Animal models have shown that early-life stressors, as well as chronic stress in adulthood, induce methylation of *BDNF* promoters I and IV leading to lower expression levels of *BDNF* in the hippocampus [65,66]. 

However, human studies have yielded conflicting results with respect to the methylation levels of *BDNF* following exposure to stress, suggesting the involvement of compensatory mechanisms (such as medication) that may reverse this effect [67]. Moreover, reduction in the acetylation levels of histone 3 (H3) at the promoters regions of BDNF following exposure to stress acts synergistically with methylation changes to downregulate *BDNF* expression in the rat hippocampus [68].

Epigenetic changes in response to exposure to stress in early life have also been documented in the human serotonin transporter (*5-HTT*), which is encoded by *SLC6A4* gene and is considered to be a key regulator of the serotoninergic system that regulates the stress response [69]. In particular, early life adversities, such as childhood abuse, have been associated with increased methylation status of *SLC6A4* at exon 1 [70,71]. However, other studies failed to replicate this finding [72] or reported hypomethylation of *SLC6A4* gene [73]. In addition, higher methylation levels of *SLC6A4* in response to stress have been reported in carriers of 5-HTTLPR S (Short) allele, a polymorphism in the promoter region that confers susceptibility to stress [73]. The functional consequence of *SLC6A4* methylation on mRNA levels appears to be multifactorial and possibly confounded by the presence of 5-HTTLPR polymorphism, given that the methylation status of *SLC6A4* has not always been linked with HTT expression [74,75].

Interestingly, epigenetic studies have demonstrated sex-dimorphic long-lasting methylation changes in various CpG sites of the oxytocin receptor gene (*OXTR)* following early-life adverse events [76]. Specifically, exposure to adverse events during childhood and adolescence has been associated with higher methylation levels of CpG sites in *OXTR* gene only in females but not in males [76]. Therefore, females appear to be more prone to epigenetic changes in *OXTR* following stress events than males, which may reflect the sex-dependent effect of oxytocin on stress responsivity. Moreover, hypermethylation of CpG sites at *OXTR* gene has been reported in patients with anorexia-nervosa [77], as well as in patients with autism spectrum disorders (ASD) [78] compared with healthy control subjects. On the other hand, reduced levels of *OXTR* methylation in the cord blood of newborns has been associated with maternal depression symptoms in the second trimester of pregnancy [79]. Moreover, luciferase reporter assays have demonstrated that the methylation status of a CpG island, which is located in the first intron, regulates the transcriptional activity of *OXTR* [80]. Summary of the most recent findings about epigenetic modifications upon stress exposure are shown in Table 1.

## 5. Epigenetic Changes and Stress-Related Disorders

Epigenetic modifications constitute part of a mechanism through which stressful life experiences are embedded in an individual’s biology, shaping the response to future threats [46]. More importantly, epigenetic changes have the intrinsic feature to persist over time, with a growing number of studies suggesting that they may predispose an individual to the development of stress-related phenotypes and diseases [46]. The causal link between epigenetic signatures and stress-related phenotypes has been unveiled by studies examining: (a) differences in methylation patterns of patients compared to controls, usually measured in peripheral tissues; and (b) if the observed differences in methylation status represent epigenetic signatures of stressful experiences occurring in the past.

Indeed, active and long-lasting demethylation of *FKBP5* gene associated with childhood abuse has been involved in the development of major depressive disorder [96]. Critically, the degree of the environmentally induced epigenetic changes has been shown to be influenced by the presence of risk alleles in *FKBP5* gene that confer a higher risk for psychiatric disorders [96]. The interplay between genetic background-epigenetic environment seems to be more relevant during critical developmental periods where the epigenome shows heightened plasticity, such as in childhood, adolescence, or later life, since no association among them has been reported during adulthood [96].

Significant attention has been given to epigenetic modifications occurring in *NR3C1* in response to environmental stressors, and their association with stress-related disorders. Studies exploring methylation changes in *NR3C1*, have investigated the effect of life-stress experiences occurring at different time periods, from as early as prenatal life to childhood and adulthood [97]. The majority of these studies reported significant hypermethylation at the exon 1F promoter of *NR3C1*, which was also positively associated with the severity and the duration of an adverse event [98]. Subsequently, several studies showed that *NR3C1* methylation is associated with reduced levels of *NR3C1* expression and with a lower number of GRs in the hippocampus [99]. It has been proposed that *NR3C1* hypermethylation act as a mediator of the association between early life adversity and stress-related disorders [98]. In particular, prenatal stress due to maternal depression and early prenatal loss has been associated with hypermethylation of CpG sites from 35 to 37, at hGR 1F promoter, triggering the development of depression, bipolar disorder and suicidal behavior later in life [100]. 

In contrast, a significant hypomethylation pattern at hGR promoters 1B and 1C has been observed in patients with post-traumatic stress disorder (PTSD) compared to controls [98]. A possible explanation for this discrepancy is that the timing of stress event may determine the direction of the effect, with adversities occurring early in life to promote hypermethylation of *NR3C1* and inhibition of hGR expression, and those appearing late in life to instigate demethylation of the same regions.

Recent studies in animal models of stress and depression revealed a decrease in Bdnf hippocampal mRNA levels, mediated by long-lasting methylation and acetylation changes in the promoter regions of *Bdnf* that were induced from perinatal exposure to methylmercury [101] or chronic social defeat stress conditions [102,103]. Moreover, in patients with depression, there was a significant decrease in BNDF protein levels compared to controls [104], while administration of antidepressants, such as selective serotonin reuptake inhibitors, stimulated *BDNF* expression and reversed the symptoms of depression [105]. Methylation-induced inhibition of *BDNF* has also been demonstrated in a study that examined genetic expression changes in the brains of suicide victims [105]. Furthermore, prevailing evidence supports higher methylation levels and decreased expression of *BDNF* in patients with schizophrenia, a fact that may explain the impaired GABAergic signaling and the reduced hippocampal volume observed in this cohort compared to controls [106]. Mice carrying the rs6265, p.Val148Met minor variant (T), exhibit lower methylation levels of *BDNF* following exposure to stress compared with controls subjects, because the G to A substitution abolishes a CpG site that is present in the wild type codon [107]. This finding was also shown in patients with schizophrenia, where Met carriers (CT or TT) were had *BDNF* hypomethylation compared to noncarriers (CC), suggesting that an individual’s genotypic background modulates the stress-induced epigenetic changes that may confer vulnerability to schizophrenia [108].

Major depressive disorder has also been associated with increased methylation levels of *SLC6A2* [109] in subjects with a history of exposure to stress early in life, such as childhood maltreatment (CM) [71] or low socioeconomic status (SES) during adolescence [110]. In particular, greater methylation levels of CpG sites 11 and 12 of SLC6A2 gene were reported in individuals with a CM history [71] and greater methylation levels of *SLC6A2* promoter were reported in individuals with a history of low SES during adolescence [110]. Both methylation patterns were associated with the emergence of depressive symptoms later in life [71,110]. As a potential mechanism, it has been proposed that reduced levels of SLC6A2 lead to dysfunction of serotonergic system by inhibiting serotonin uptake, which in turn may increase the susceptibility to depression [111]. 

An interesting interplay between genetic variation and epigenetic regulation in response to stress was shown in the study of Lei et al., where the methylation status of *SLC6A2* correlated positively with depression symptoms only in individuals carrying the short risk allele of *SLC6A2* [112]. However, the study of Alassari et al. [73] that examined the epigenetic effect of work-related stress on *SLC6A2* methylation status revealed opposite results, with the high-stress environment being associated with hypomethylation of *SLC6A2*. As previously outlined, a possible explanation for this discrepancy is that the timing of stress exposure may dictate the direction of the effect, with stressors appearing early in life (CM, SES during adolescence) to promote hypermethylation of *SLC6A2* and stressors occurring during adulthood to promote hypomethylation of *SLC6A2*.

The OXTR protein plays an important role in the regulation of social cognition (feelings of attachment and social recognition), as well as in the regulation of anxiety-related and social behaviors [79]. In view of its effect, it has been proposed to administrate intranasally OXT in individuals with social-affective deficits or psychiatric disorders [113]. In female subjects, adverse events in adulthood (financial pressure, high crime neighborhood) may induce methylation pattern changes in *OXTR* and may increase vulnerability to depression [113]. Furthermore, early traumatic events in childhood have been associated with altered methylation status of *OXTR* that may mediate the development of mood disorders in adulthood [114]. More specifically, hypomethylation of CpGs located in the promoter region of *OXTR* exon 1 and hypermethylation of sites located in *OXTR* intron 3 have been associated with depression and anxiety disorders in subjects with a history of childhood abuse [114].

A summary of the most recent findings about epigenetic modifications in patients with stress-related disorders are shown in Table 2.

## 6. Epigenetic Effects of Stress Exposure: Challenges and Confounders

Studies investigating the epigenetic effects of stress experiences on the methylation status of stress-related genes sometimes report conflicting results about the direction of epigenetic effect (hypomethylation or hypermethylation) or fail to replicate their findings. A possible explanation for these discrepancies may be the highly dynamic and spatiotemporal nature of the epigenetic landscape, suggesting that several factors and features of stress response should be taken into account in the design of epigenetic studies to ensure comparative results [46,128]. 

First, methodological variables related to stressor characteristics, such as the type, the duration and the timing of the stressful event, should be controlled and normalized across different studies to minimize background noise derived from the magnitude of the stress stimulus and the context-dependent conceptualization of threat that orchestrates the stress response [46]. Furthermore, the individual’s characteristics, such as age, gender and genetic composition, may account for differences in epigenetic studies, as we have seen in the case of *OXTR* methylation status in females vs. males [76] and *SLC6A4* methylation status in the presence of 5-HTTLPR S short allele [73]. 

Methodological differences between studies, including the tools stratified to measure certain types of adversities and the type of the study, cross-sectional vs. longitudinal, may account for divergent findings and are issues that should be addressed in future human epigenetic research [69]. In addition, the context-dependent nature of epigenetic modifications should be taken into account, given that epigenetic effects of stressors may not be the same across different tissues and may differ in peripheral blood versus tissues relevant to the stress-related disorders upon investigation [46,129].

## 7. Conclusions

In this article, we present existing knowledge about epigenetic changes occurring in response to stress and their association with the emergence of stress-related disorders, focusing on GCs as primary mediators of these effects. Compelling evidence suggests that epigenetic modifications in stress-related genes involved in glucocorticoid signaling represent a mechanism through which stress-related experiences are embedded in an individual’s biology. Epigenetic changes can influence the subsequent coping strategies [122] to stressors and, when accumulated, can contribute to the development of stress-related disorders [46]. A number of studies, claimed that epigenetic changes can actually be inherited to the next generation and as such parent’s stressful experiences could influence offspring’s vulnerability to many pathological conditions [57,130,131,132]. However, many researchers are sceptical about the potential of trauma inheritance through epigenome [133] and also highlight the need to perform epigenetic studies taking into account an individual’s genetic background [134,135].

The imperfect fit of different epigenetic studies that follow a traditional reductionist paradigm, suggests that the interaction of stressors with the epigenome and its implication in the emergence of stress-related disorders is a much more complex network than previously thought, and demands our investigation in a context-specific and time-dependent manner. We hope that this article will help future studies, to build upon existing and novel findings their design, taking into account confounders that may bias study results, in order to delineate the mechanism linking stress exposure to epigenome and stress-related disorders development via the action of GCs.

## Figures and Tables

**Table 1:** Summary of the most recent findings about epigenetic modifications upon stress exposure.

Gene	Reference	Species	Stressors	Epigenetic Changes
*FKBP5*	Saito et al., 2020 [81]	Human	Childhood abuse	demethylation of FKBP5 intron 7
	Misiak et al., 2020 [82]	Human	Adverse childhood experiences (ACEs)	demethylation of FKBP5
	Ramo-Fernandez et al., 2019 [83]	Human	Childhood maltreatment	demethylation of FKBP5
*NR3C1*	Borcoi et al., 2021 [84]	Human	Food and nutritional security or insecurity status	hypermethylation of NR3C1 1F promoter
	Pinheiro et al., 2021 [85]	Human	Alcohol consumption, Body mass index—BMI	hypomethylation of NR3C1 1F promoter (alcohol consumption), hypermethylation of NR3C1 1F promoter (BMI)
	Misiak et al., 2021 [86]	Human	Adverse childhood experiences	hypomethylation of NR3C1
*BDNF*	Duffy et al., 2020 [87]	Rat	Aversive caregiving	hypermethylation BDNF
	Blaze et al., 2017 [88]	Rat	Caregiver maltreatment	hypermethylation BDNF IV
	Niknazar et al., 2017 [89]	Rat	Preconception maternal stress	hypermethylation BDNF
*SLC6A4*	Delano et al., 2021 [90]	Human	Maternal community-level deprivation	hypermethylation of 8 CpGs SLC6A4
	Anurag et al., 2019 [91]	Human	Early adversity (children of alcoholics)	hypermethylation of SLC6A4
	Smith et al., 2017 [92]	Human	Adverse neighborhood environment	hypermethylation of SLC6A4
*OXTR*	Kogan et al., 2019 [93]	Human	Childhood adversity and socioeconomic instability	hypermethylation of OXTR
	Kogan et al., 2018 [94]	Human	Childhood adversity	hypermethylation of OXTR
	Gouin et al., 2017 [95]	Human	Early life adversity (ELA)	hypermethylation of OXTR in females

**Table 2:** Summary of the most recent findings about epigenetic modifications in stress-related disorders.

Gene	Reference	Species	Stress-related disorder	Findings
*FKBP5*	Mihaljevicab et al., 2021 [115]	Human	Psychotic disorder	(A) demethylation of FKBP5 (B) genetic background influence epigenetic modifications
	Kang et al., 2018 [116]	Human	Post-Traumatic Stress Disorder (PTSD)	(A) demethylation of FKBP5 (B) genetic background influence epigenetic modifications
	Klinger-Konig et al., 2019 [117]	Human	Depression	(A) demethylation of FKBP5 associated with depression (B) genetic background influence epigenetic modifications
	Misiak et al., 2020 [82] *	Human	Psychotic disorders	(A) demethylation of FKBP5 associated with depression
*NR3C1*	Borcoi et al., 2021 [84] *	Human	Depressive symptoms	(A) hypermethylation of NR3C1 1F promoter
	Pinheiro et al., 2021 [85] *	Human	Depression	(A) hypermethylation of NR3C1 1F receptor
	Bakusic et al., 2021 [118]	Human	Depression	(A) hypermethylation of NR3C1
	Misiak et al., 2021 [86] *	Human	Various stages of psychosis	(A) hypomethylation of NR3C1 in first-episode psychosis patients (B) hypermethylation of NR3C1 in schizophrenia patients
*BDNF*	Guo et al., 2021 [119]	Human	Post-Traumatic Stress Disorder (PTSD)	(A) hypermethylation of BDNF promoter (B) genetic background influence epigenetic modifications
	Hossack et al., 2020 [120]	Human	Post-Traumatic Stress Disorder (PTSD)	(A) hypomethylation of BDNF promoter
	Peng et al., 2019 [121]	Human	Depression	(A) hypermethylation of BDNF promoter at 2 CpGs (B) hypomethylation of BDNF promoter at 1 CpG
	Shirata et al., 2020 [122]	Human	High sociotropy	(A) hypermethylation of BDNF promoter
*SLC6A4*	Sanwald et al., 2021 [123]	Human	Depression	(A) hypermethylation of SLC6A4
	Hossack et al., 2020 [120]	Human	Post-Traumatic Stress Disorder (PTSD)	(A) hypomethylation of SLC6A4
	Peng et al., 2019 [121]	Human	Depression	(A) hypermethylation of SLC6A4
	Schneider et al., 2017 [124]	Human	Major Depression Disorder (MDD)	(A) hypomethylation of SLC6A4
*OXTR*	Nawjin et al., 2018 [125]	Human	Post-Traumatic Stress Disorder (PTSD)	(A) hypermethylation of OXTR in females
	Kogan et al., 2018 [94]	Human	Substance use problem	(A) hypermethylation of OXTR
	Ein-Dor et al., 2018 [126]	Human	Attachment avoidance	(A) hypermethylation of OXTR
	Cappi et al., 2016 [127]	Human	Obsessive-compulsive disorder	(A) hypermethylation of OXTR

* Methylation status was also associated with previous stress-experience, see Table 1.

## Data Availability

Data sharing not applicable.
